# Seroprevalence of Zika virus and dengue virus infections in migrants in Italy

**DOI:** 10.3389/fcimb.2025.1617029

**Published:** 2025-07-10

**Authors:** Federica Frasca, Francesco Eugenio Romani, Elio Gentilini Cacciola, Francesca Colavita, Enrico Palermo, Luca Maddaloni, Luigi Rosa, Alessandra D’Auria, Valentina Baccolini, Giuseppe Migliara, Giulia Matusali, Giancarlo Ceccarelli, Guido Antonelli, Fabrizio Maggi, Gabriella d’Ettorre, Carolina Scagnolari

**Affiliations:** ^1^ Department of Public Health and Infectious Diseases, Sapienza University of Rome, Rome, Italy; ^2^ Laboratory of Virology, Department of Molecular Medicine, Sapienza University of Rome, Rome, Italy; ^3^ Laboratory of Virology, National Institute for Infectious Diseases “Lazzaro Spallanzani” IRCCS, Rome, Italy; ^4^ Istituto Pasteur Italia-Cenci Bolognetti Foundation, Viale Regina Elena 291, Rome, Italy; ^5^ Department of Life Sciences, Health and Health Professions, Link Campus University, Rome, Italy; ^6^ Microbiology and Virology Unit, Policlinico Umberto I Hospital, Sapienza University of Rome, Rome, Italy; ^7^ Department of Molecular Medicine, Sapienza University of Rome, Laboratory Affiliated to Istituto Pasteur Italia-Fondazione Cenci Bolognetti, Rome, Italy

**Keywords:** zika virus, dengue virus, seroprevalence, migrants, North Africa, sub-Saharan Africa, south-east Asia, orthoflavivirus Zika virus

## Abstract

**Introduction:**

Estimating the burden of Zika virus (ZIKV) and dengue virus (DENV) infections in migrants is important to promote their health status and recommend appropriate interventions. We aimed to estimate the seroprevalence of ZIKV and DENV in migrants from high endemic countries attended at a referral center in Rome (Italy), arriving via the Mediterranean from North and sub-Saharan Africa and South-East Asia.

**Methods:**

Sixty-four serum samples from migrants were tested for anti-ZIKV and anti-DENV immunoglobulin (Ig) G and IgM by ELISA. Serum samples with detectable Ig were analyzed by indirect immunofluorescence assay (IFA). For confirmatory testing and given the cross-reactivity of antibodies between orthoflaviviruses, all positive IFA sera were tested by virus neutralization test. ZIKV and DENV RNA were assessed by RT Real-Time PCR.

**Results:**

All serum samples were negative for anti-ZIKV IgG, while 12.50% (n=8/64) were positive for anti-ZIKV IgM by ELISA. IFA showed that only 1 of 8 serum samples (12.50%) was positive for anti-ZIKV IgM, but ZIKV RNA was undetectable. The seroprevalence of anti-DENV IgG by ELISA was 59.37% (n=38/64), mostly confirmed by IFA (97.36%, n=37/38). Furthermore, anti-DENV IgM were detected in 9 serum samples (n=9/64, 14.06%) by ELISA, previously tested negative for anti-DENV IgG. Of these, 2 sera were confirmed by IFA, but DENV RNA was not detectable. Anti-DENV neutralizing antibodies (nAbs) were detected in 27% of anti-DENV IgG sera (n=10/37) tested by IFA. Multiple linear regression analysis showed that sub-Saharan African origin was an independent factor for the development of anti-DENV nAbs (p=0.009), while age and gender had no effect. Sera negatives for anti-DENV nAbs but with detectable anti-DENV IgG tested by IFA had nAbs to another orthoflavivirus (n=25/27, 92.59%) such as West Nile virus (WNV) (n=17/25, 68%), Yellow fever virus (YFV) (n=7/25, 28%) and Usutu virus (USUV) (n=1/25, 4%).

**Discussion:**

A high prevalence of anti-orthoflavivirus IgG, especially against DENV, was found in the migrant population studied, but no infections were detected. With the recent outbreaks of autochthonous DENV infections in Italy, the risk of secondary DENV infection - and severe disease – could be high. Robust serological surveillance, vaccination and prevention strategies for this vulnerable group are needed.

## Introduction

In recent decades, numerous infectious disease outbreaks, including the COVID-19 pandemic and the global burden of arboviruses, have profoundly affected societies worldwide (GBD 2013 DALYs and HALE Collaborators et al., 2015; Alizadeh et al., 2023). At the same time, the 21st century has seen unprecedented technological advances and significant demographic changes (Baker et al., 2022). These include rapid population growth, increased air travel and increased rural-urban migration (Baker et al., 2022). Remarkably, the increase in migratory flows towards the central Mediterranean route, with Italy as the main entry point from sub-Saharan Africa to Europe, followed in recent years by South-East Asia, is raising renewed concerns about management issues and the potential risks to local populations (Governo Italiano – Ministero dell’Interno, 2022; United Nations, Department of Economic and Social Affair, Population Division, 2016). While there are pan-European plans to address tuberculosis, HIV, viral hepatitis and vaccine-preventable diseases, the health of migrants has often been overlooked, hindering progress towards infectious disease control and vaccination targets (Baggaley et al., 2022). Indeed, there is still considerable debate on how to optimize health systems to better serve migrant populations, highlighting the need to reassess and develop effective, evidence-based infectious disease services tailored to their needs (Pareek et al., 2018). In particular, migrant’s limited access to health care, both during transit and upon arrival in Italy, may limit early detection and treatment of infectious diseases, including those caused by emerging pathogens such as mosquito-borne orthoflaviviruses, potentially worsening health outcomes and facilitating widespread transmission (Tramuto et al., 2024). Among the orthoflaviviruses responsible for a variety of potentially serious human diseases, dengue virus (DENV) causes plasma leakage and abnormal hemostasis (Pierson and Diamond, 2020), while Zika virus (ZIKV) causes congenital neurological disorders and is associated with Guillain-Barré syndrome (Pierson and Diamond, 2020). The epidemiological patterns of both viruses are alarming. DENV is endemic in more than 100 countries in Africa, the Americas, South and South-East Asia, and the Western Pacific, causing 100–400 million DENV cases and more than 9000 deaths each year (Stanaway et al., 2016; Bhatt et al., 2013). In parallel, an increasing number of autochthonous DENV cases have been described in several European countries in recent years, with Italy reporting more than 200 cases in 2024 (Frasca et al., 2024; Del Manso et al., 2024). The closely related ZIKV caused several outbreaks in tropical Africa and South-East Asia until 2007. Subsequently, in 2016, a public health emergency of international concern was declared for the ZIKV epidemic, which rapidly spread to nearly 50 countries and territories in the Americas after being detected in Brazil in 2015 (Caminade et al., 2017; Zhang et al., 2017). As the epidemiological situation is constantly evolving, seroprevalence surveys can be used as a population surveillance strategy for ZIKV and DENV to guide public health interventions. Moreover, the true burden of these infections remains underestimated in sub-Saharan Africa and South-East Asia, especially in rural and remote areas, due to the limited capacity to conduct effective and sustained surveillance of arboviruses and vectors (Bangoura et al., 2024; Perng et al., 2019). Efforts should therefore focus not only on reducing the importation of arboviruses from abroad, but also on preventing further spread of autochthonous cases. Specific measures should be taken to identify and protect at-risk populations, particularly those with underlying medical conditions or socio-economic barriers to adequate access to health care and assistance, such as migrants.

On the light of the aforementioned considerations, this was the first study to assess the seroprevalence of ZIKV and DENV in migrants arriving in Italy via the Mediterranean routes from North and sub-Saharan Africa and South-East Asia, where these orthoflaviviruses are considered endemic (Asaga et al., 2023), in order to gain additional insight into the circulation of ZIKV and DENV in their countries of origin and the transmission dynamics in relation to migration routes and flows.

## Materials and methods

### Study design and sampling

**Figure 1:**
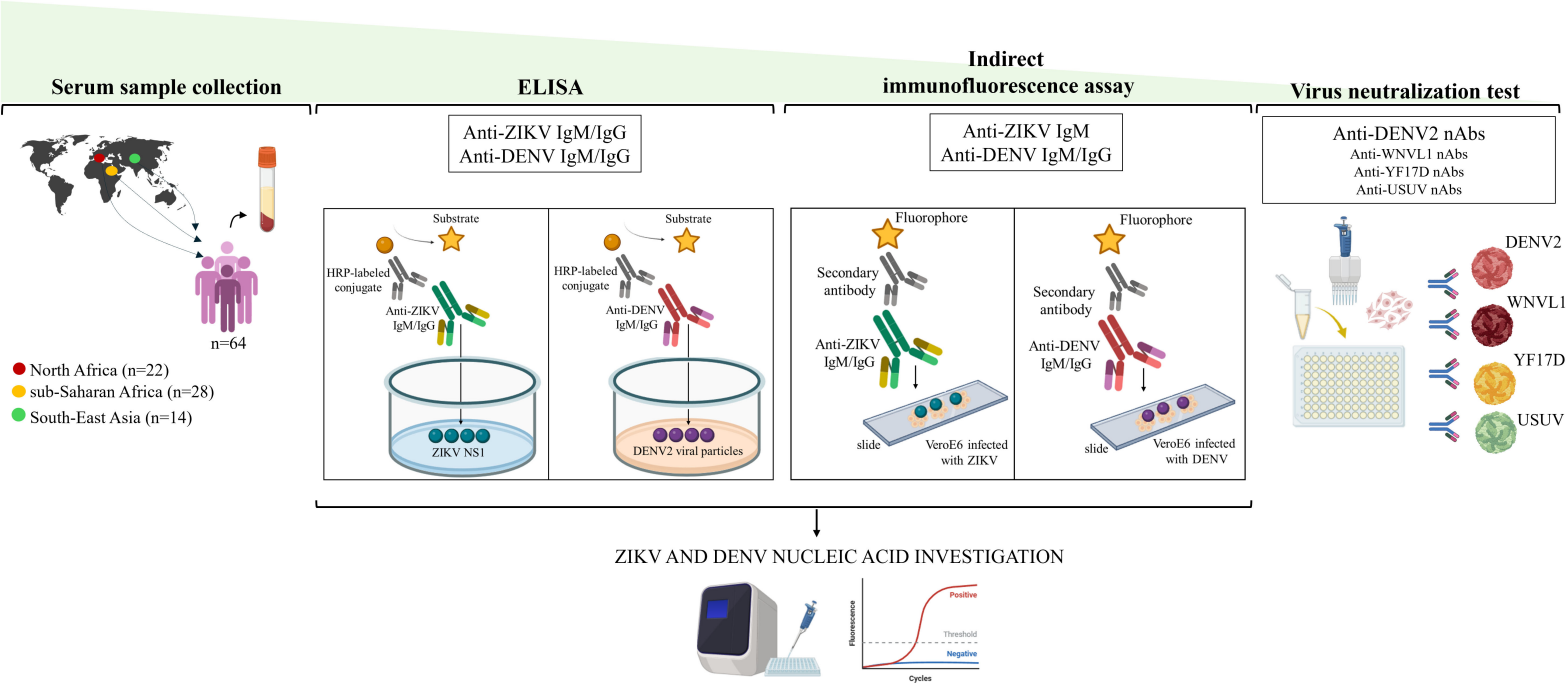
Study Pipeline. ZIKV, Zika virus; DENV, dengue virus; HRP, horseradish peroxidase; NS1, non structural protein 1; WNVL1, West Nile Virus lineage 1; YF17D, yellow fever 17D; USUV, Usutu virus; nAbs, neutralizing antibodies.

A retrospective study was conducted over a 6-month period from January 2023 to June 2023, at the Migration Outpatient Service of the Policlinico Umberto I hospital in Rome, Italy (**Figure 1**). Blood samples were collected from sixty-four newly arrived migrants from different parts of African and Asian countries. Sociodemographic characteristics (age, sex and country of origin) were recorded at the time of sample collection. Our study was outlined by eligibility criteria that were common to all enrolled participants. Inclusion criteria were as follows: i) all individuals who gave informed consent before the start of the study; ii) male and female adults ≥ 18 years of age; iii) participants for whom ZIKV and DENV are endemic in their country of origin. Exclusion criteria were as follows: i) failure to sign the informed consent; ii) contraindications to blood sampling. Blood samples were collected in non-anticoagulant tubes and transported to the laboratory in a freezer within 2–4 hours of collection. The blood samples were then centrifuged at 1800 rpm for 10 minutes to separate the sera. Serum samples were stored at -20°C. Ethical approval for this study was obtained from the Ethics Committee of the Policlinico Umberto I, Sapienza University of Rome.

### Zika and dengue IgM and IgG detection by ELISA

Serum samples from migrants were initially screened for ZIKV and DENV antibodies by using commercially available immunoglobulin (Ig) M and IgG capture ELISA kits according to the manufacturer’s instructions (**Figure 1**). ELISA plates for anti-ZIKV IgM and IgG were coated with non-structural protein 1 (NS1) of ZIKV (NovaLisa^®^ Zika Virus IgM capture ELISA; NovaLisa^®^ Zika Virus IgG capture ELISA, Szabo-Scandic HandelsgmbH, Austria). The ELISA assays for anti-DENV IgM and IgG, were prepared using a highly purified preparation of DENV2 particles (Novalisa Dengue Virus IgM ELISA- DENM0120, Szabo-Scandic HandelsgmbH, Austria; Dengue Virus IgG ELISA - DENG0120, Demeditec Diagnostics GmbH, Germany). Current or recent infection (IgM) for ZIKV and DENV and past infection (IgG) were defined as IgM or IgG titers >11 units (U), according to the manufacturer’s instructions. All equivocal samples with antibody titers between 9 and 11 U were retested and if the results were again uncertain, the specimens were considered negative. Serum samples with antibody titers < 9 were considered negative.

### Zika and dengue IgM and IgG detection by IFA

An in-house indirect immunofluorescence assay (IFA) was used to further analyze serum samples with anti-ZIKV and anti-DENV antibody titers >11 U determined by ELISA (Colavita et al., 2019) (**Figure 1**). Briefly, IgG and IgM were detected on slides prepared in-house with VeroE6 cells infected with ZIKV (INMI1/Brazil-2016 isolate strain, Ref-SKU: 009V-00880) and DENV [serotype 2, New Guinea-C strain, dengue virus 2 (NCPV 0006041v)] isolates, as previously described (Colavita et al., 2019). ZIKV and DENV home-made slides were preliminary tested with three negative and three positive control samples at different 2-fold dilutions (from 1:2 to 1:40). The 1:20 dilution resulted in the best conditions for eliminating the background signal and obtaining clear positive fluorescence with good sensitivity. Hence, all sera were pre-treated using Eurosorb reagent (Euroimmun, Lubecca, Germany) and tested at serial dilutions from 1:20 down to 1:1280 to estimate the antibodies titers. FITC-conjugated anti-human IgM and IgG antibodies (Euroimmun, Lubecca, Germany) were used as secondary antibodies and Evans Blue as cell counterstain. Results were analyzed using a Nikon Eclipse E600 fluorescence microscope. Each experiment included positive controls with anti-ZIKV and anti-DENV antibodies and negative control. The positive controls used for the IFA were obtained from patients with confirmed DENV or ZIKV infection, for whom follow-up serum samples were available.

### Virus neutralization tests

Serum samples that were positive for anti-DENV antibodies by the IFA test were subjected to the virus neutralization test (VNT) for confirmatory testing as previously described (Colavita et al., 2020) (**Figure 1**). As the antibody response generated against orthoflavivirus infection is cross-reactive, we also tested serum samples in the VNT for cross-reactivity to other orthoflaviviruses (yellow fever, West Nile and Usutu viruses). The virus strains used for VNT were: DENV2 (New Guinea-C strain, NCPV 0006041v), yellow fever virus (YFV, vaccine strain 17D), Usutu virus (USUV) (Scagnolari et al., 2013), West Nile virus lineage 1 (WNVL1) (Cacciotti et al., 2015). Sera were heat-inactivated, diluted 1:10, and titrated in duplicate at two-fold dilutions. Equal volumes of 100 TCID_50_/well of virus and serum dilutions were mixed and incubated at 37°C for 30 minutes. VeroE6 cells seeded in sub-confluent 96-well plates were then incubated with 100 µL/well of each orthoflavivirus-serum mixture at 37°C, 5% CO_2_. The highest serum dilution that inhibited at least 90% of the cytopathic effect (CPE) was defined as positive for the presence of neutralizing antibodies (nAbs). nAbs were considered undetectable if titers were <1:10. Positive and negative neutralization controls were included in each experiment.

### Detection of Zika virus and dengue virus RNA

Sera from migrants with detectable anti-ZIKV and anti-DENV IgM by ELISA and IFA were further tested by RT Real-Time PCR assay for the detection of the polyprotein gene of ZIKV and DENV, respectively (**Figure 1**). Briefly, RNA was extracted from 200 µL of serum samples using a Total RNA Purification Kit (Norgen Biotek Corp, Thorold, Ontario, Canada). The concentration and purity of 2 µL of purified RNA were assessed using a NanoDrop Spectrophotometer (Thermo Fisher Scientific, Waltham, Massachusetts). Reverse transcription was then performed on 300 ng of purified RNA using the method described previously (Santinelli et al., 2019). The housekeeping gene 18S ribosomal RNA (rRNA) was used as an internal control to assess the efficiency of cDNA synthesis. A gene transcript was considered not detectable when the threshold cycle (Ct) value was > 40 and was assigned a value of one tenth of the limit of detection. Primer pairs for ZIKV (Forward: 5’-AARTACACATACCARAACAAAGT-3’; Reverse: 5’-TCCRCTCCCYCTYTGGTCTTG-3’) were added to Master Mix (QuantiTect SYBR Green PCR Kit-Qiagen; Hilden, Germany) at 50 µM in a final reaction volume of 10 µL. Primers and probes for DENV (Probe: 5’-FAM-AGCATCATTCCAGGCAC-TAM-3’; Forward: 5’-ACCATTCCATTTTCTGGCGTT-3’; Reverse: 5’-GARAGACCAGAGATCCTGCTGTCT-3’) and 18S rRNA (Probe: 5’-ACCGGCGCAAGACGGACCAGA-TAM-3’; Forward: 5’-CGCCGCTAGAGGTGAAATTCT-3’; Reverse: 5’-CATTCTTGGCAAATGCTTTCG -3’) were added to the Probes Master Mix (Roche, Basel, Switzerland) at 500 and 250 nM in a final reaction volume of 20 µL. To ensure quality control, positive and negative controls were included in each run and validated before analysing patient samples. Specifically, the positive control for ZIKV consisted of cDNA synthesized from RNA extracted after propagation of the virus in C6/36 cell cultures. For DENV, we used cDNA obtained by reverse transcription of RNA extracted from a serum sample confirmed to be positive for DENV by molecular testing. In addition, a negative extraction blank which was a known negative sample was included in each run to check for cross-contamination and specificity.

### Statistical analysis

**Figure 2:**
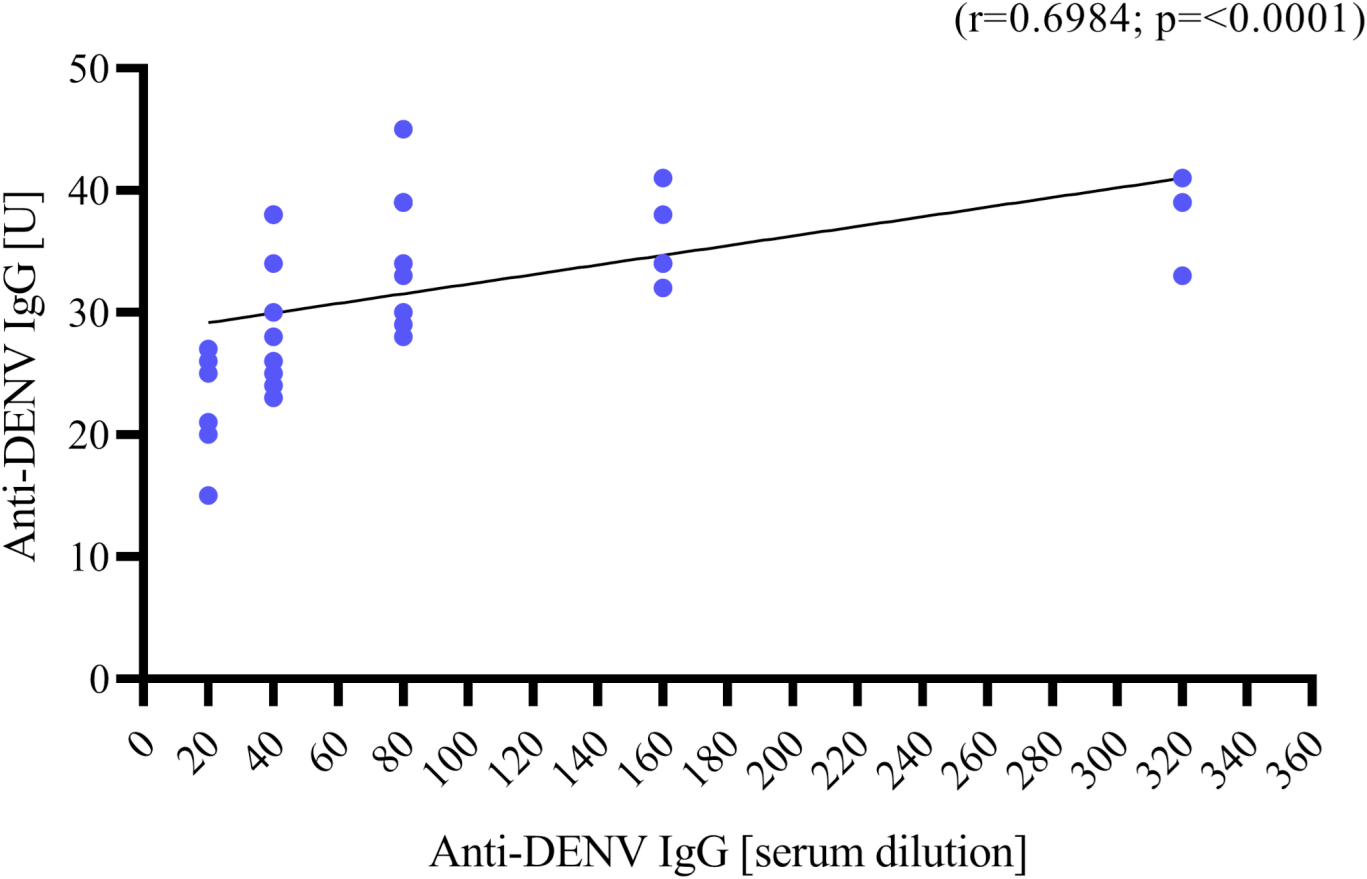
Correlation between anti-DENV IgG titer in Units [U] measured by ELISA (*y axis*) and anti-DENV IgG titer in serum dilution measured by IFA (*x axis*, starting from 1:20 to 1:320). Correlation was assessed using Spearman’s ρ coefficient.

Descriptive analysis was performed, using total number (N) and percentages (%) for categorical variables, while quantitative data were described using median and range (Min-Max). Spearman’s rho coefficient was calculated to assess the correlation between levels of anti-DENV IgG tested by ELISA and IFA. Differences in the frequencies of migrants’ countries of origin were determined using Pearson chi-squared test. To identify characteristics associated with the development of anti-DENV2 nAbs, the Pearson Chi-square test was used for dichotomous and categorical variables. A multivariable logistic regression model was built to investigate independent associations with the development of anti-DENV2 nAbs, estimating adjusted odds ratios (aORs) and 95% confidence intervals (CIs) for each characteristic of interest. The following variables were included in the multivariable model, based on expert knowledge: age (continuous), gender (0 male; 1 female), country of origins (0 Other origin; 1 sub-Saharan Africa). A two-sided p value <.05 was considered statistically significant. Data collected were analyzed using SPSS statistical software version 20 (IBM, USA) and STATA version 17.0 (StataCorp LLC, 4905 Lakeway Drive, College Station, TX, USA). Graph in **Figure 2** was made using Graph Pad PRISM 8.0.1 software.

## Results

### Participants characteristics

A total of sixty-four serum samples from migrants were included in the study. The demographic characteristics and country of origin of migrants are described in **Table 1**. The median age of the migrants was 27 years (range: 18 - 46). There were 56 men (87.50%) and 8 women (12.50%). The most common continent of origin was Africa [n=50/64 (78.12%)], mainly North [n=22/50 (44%)] and sub-Saharan Africa [n=28/50 (56%)], followed by South-East Asia [n=14/64 (21.87%)] (**Table 1**). The frequency of migrants from Africa was higher than that of migrants from South-East Asia [n=50/64 (78.12%) migrants from Africa vs n=14/64 (21.87%) migrants from South-East Asia, p<0.0001].

**Table 1:** Demographic characteristics and origins of migrants.

Items	Migrants	Males	Females
Total number	64	56	8
Age (range), years	27 (18 - 46)	28 (18 - 46)	27 (18 - 29)
Africa*,**			
North Africa [N, (%)]	22/64 (34.37)	21/56 (37.50)	1/8 (12.50)
sub-Saharan Africa [N, (%)]	28/64 (43.75)	22/56 (39.28)	6/8 (75)
Asia*			
South-East Asia [N, (%)]	14/64 (21.87)	13/56 (23.21)	1/8 (12.50)

Data are expressed as total number (N) and percentage (%). Age range is indicated as median (Min-Max).

*North Africa countries: Egypt (n=18), Morocco (n=1), Tunisia (n=2); Palestine (n=1); sub-Saharan Africa: Burkina Faso (n=1), Cameroon (n=4), Ivory Coast (n=7), Eritrea (n=1), Ethiopia (n=1), Guinea (n=6), Mali (n=1), Nigeria (n=1), Senegal (n=1), Sierra Leone (n=1), Somalia (n=3), Togo (n=1); South-East Asia countries: Bangladesh (n=8), Iran (n=1), Pakistan (n=3), Syria (n=2). ** The frequencies of migrants from Africa were higher compared to those of migrants from South-East Asia (p<0.0001) using Pearson chi-squared test.

### Anti-Zika virus IgM/IgG and viral RNA detection

All serum samples of migrants were negative for anti-ZIKV IgG by ELISA (anti-ZIKV IgG titers <9 U). In contrast, 8 out of 64 sera (12.50%) tested positive for anti-ZIKV IgM [median titer: 18.5 U (range: 11 – 39)]. All serum samples positive for IgM to ZIKV by ELISA underwent the anti-ZIKV IgM IFA testing and Real-Time PCR for ZIKV-RNA detection. The results showed that 1 serum sample (n=1/8, 12.5%) was confirmed positive for anti-ZIKV IgM (serum dilution for anti-ZIKV IgM: 1:20) (**Table 2**). All serum samples with detectable anti-ZIKV IgM by ELISA underwent RT Real-Time PCR for 18S rRNA, and Ct values were detected [mean: 23.34, standard deviation (SD): 1.38, range: 20.85 – 24.66]. However, no detectable levels of ZIKV RNA were found.

**Table 2:** Frequencies and levels of anti-ZIKV and anti-DENV antibody titers determined by different serological assays.

Items	ELISA	Indirect immunofluorescence assay (IFA)	
			Anti-DENV2 neutralization test
Anti-ZIKV IgG+ [N, (%)] Median (Min-Max)	0 (0) NA	NA NA	NA NA
Anti-ZIKV IgM+ [N, (%)] Median (Min-Max)	8/64 (12.50) 18.5 U (11 – 39)	1/8 (12.50) 1:20	0/8 (0) NA
Anti-DENV IgG+ [N, (%)] Median (Min-Max)	38/64 (59.37) 33 U (15 - 45)	37/38 (97.36) 1:80 (1:20 - 1:320)	10/37 (27)^°^ 1:40 (1:20 - 1:160)
Anti-DENV IgM+ [N, (%)] Median (Min-Max)	9/64 (14.06) 17 U (12 – 22)	2/9 (22.22) 1:20	0/2 (0) NA

The data are expressed as the total number (N), percentage (%) and the median titer (Min-Max). Serum samples have been initially screened for ZIKV and DENV antibodies by using immunoglobulin (Ig) M and IgG capture ELISA. Subsequently, serum samples with anti-ZIKV and anti-DENV antibody titers > 11 Units (U) determined by ELISA were analyzed by in-house indirect immunofluorescence assay (IFA) and those positive for anti-DENV antibodies by the IFA test were subjected to the virus neutralization test (VNT). The titers of anti-ZIKV and anti-DENV antibodies are expressed in units for ELISA, and as serum dilution for IFA and the anti-DENV2 neutralization test. ^°^Two anti-DENV2 nAbs positive serum samples showed similar levels of nAbs to YF17D or WNV, respectively. NA=not applicable.

### Anti-dengue virus IgM/IgG and viral RNA detection

Serum samples from 38 migrants (n=38/64, 59.37%) were positive for anti-DENV IgG by ELISA with a median titer of 33 U (range: 15 – 45). The IFA results indicated that 37 samples (n=37/38, 97.36%) were confirmed positive for DENV antibodies, with heterogeneous levels of anti-DENV IgG titers [median serum dilution for anti-DENV IgG: 1:80 (range: 1:20 – 1:320)] (**Table 2**). There was a positive correlation between anti-DENV IgG levels measured by ELISA and those obtained by IFA (r=0.6984, p<0.0001) (**Figure 2**). In addition, anti-DENV IgM were detected in 9 serum samples (n=9/64, 14.06%) by ELISA [median titer 17 U (range: 12 – 22)], previously tested negative for anti-DENV IgG. Of these, 2 sera were confirmed by IFA but with a borderline titer (serum dilution of anti-DENV IgM: 1:20). All sera positive for anti-DENV IgM were further tested for 18S rRNA and DENV-RNA by RT Real-Time PCR. Ct values of 18S rRNA were detected (mean: 24.7, SD: 2.14, range: 21.92 – 29.26). However, no detectable levels of DENV RNA were found.

### Anti-DENV2 neutralization test

**Figure 3:**
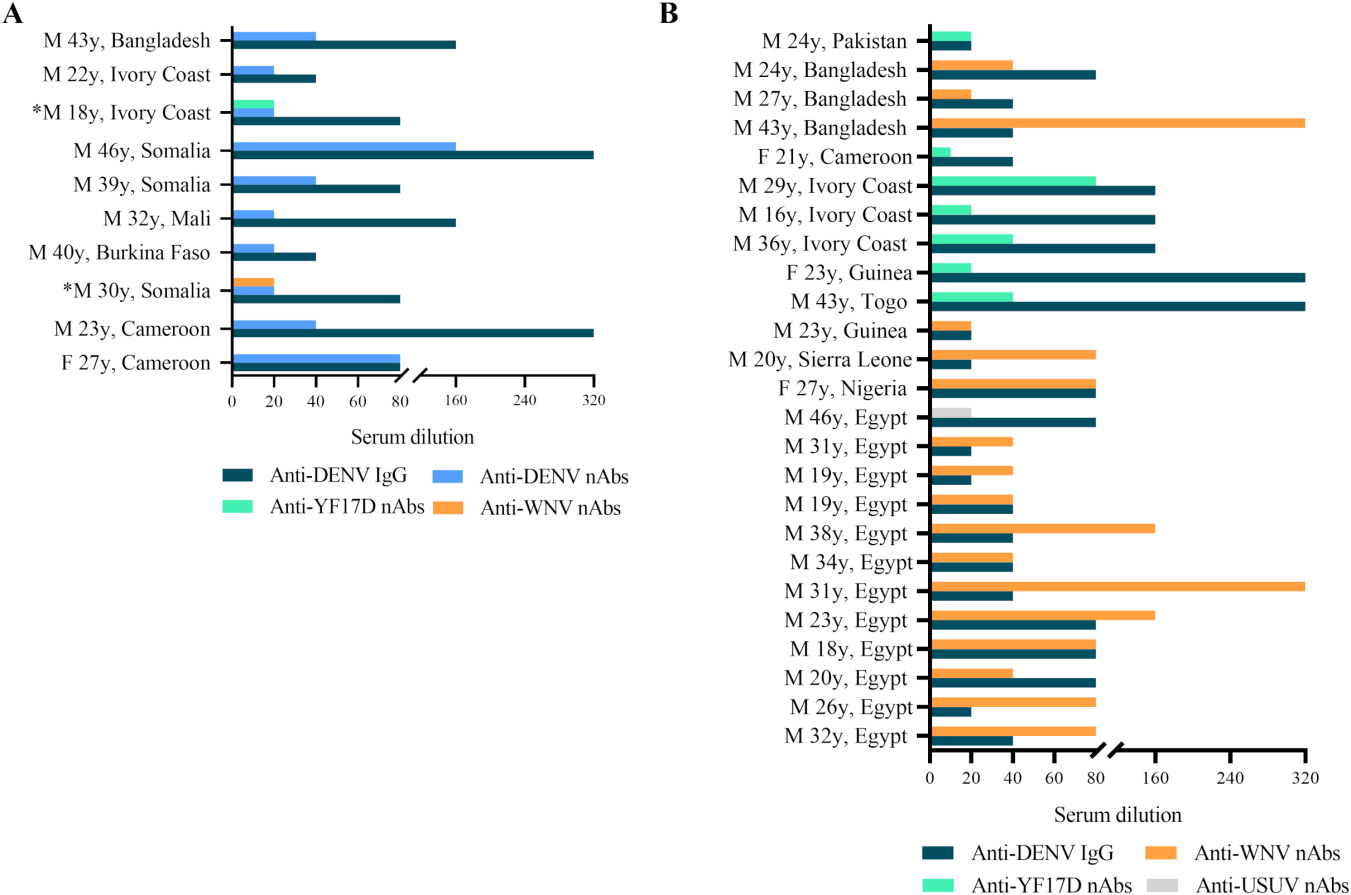
**(A)** Anti-DENV IgG titers (serum dilution, starting at 1:10, blue bar) measured by IFA and anti-DENV2 nAbs (serum dilution, starting at 1:20, light blue bar) tested by virus neutralization assay (VNT) in serum samples from migrants (n=10); *Serum samples had similar levels of nAbs against anti-DENV2 and another orthoflavivirus. **(B)** Titers of anti-DENV IgG (serum dilution, starting at 1:10) measured by IFA and anti-WNV nAbs (serum dilution, starting at 1:20, orange bar), anti-YF17D nAbs (serum dilution, starting at 1:20, green bar) and anti-USUV nAbs (serum dilution, starting at 1:20, grey bar) measured by VNT in anti-DENV2 nAbs negative but IgG positive serum samples by ELISA/IFA tests (n=25).

**Figure 4:**
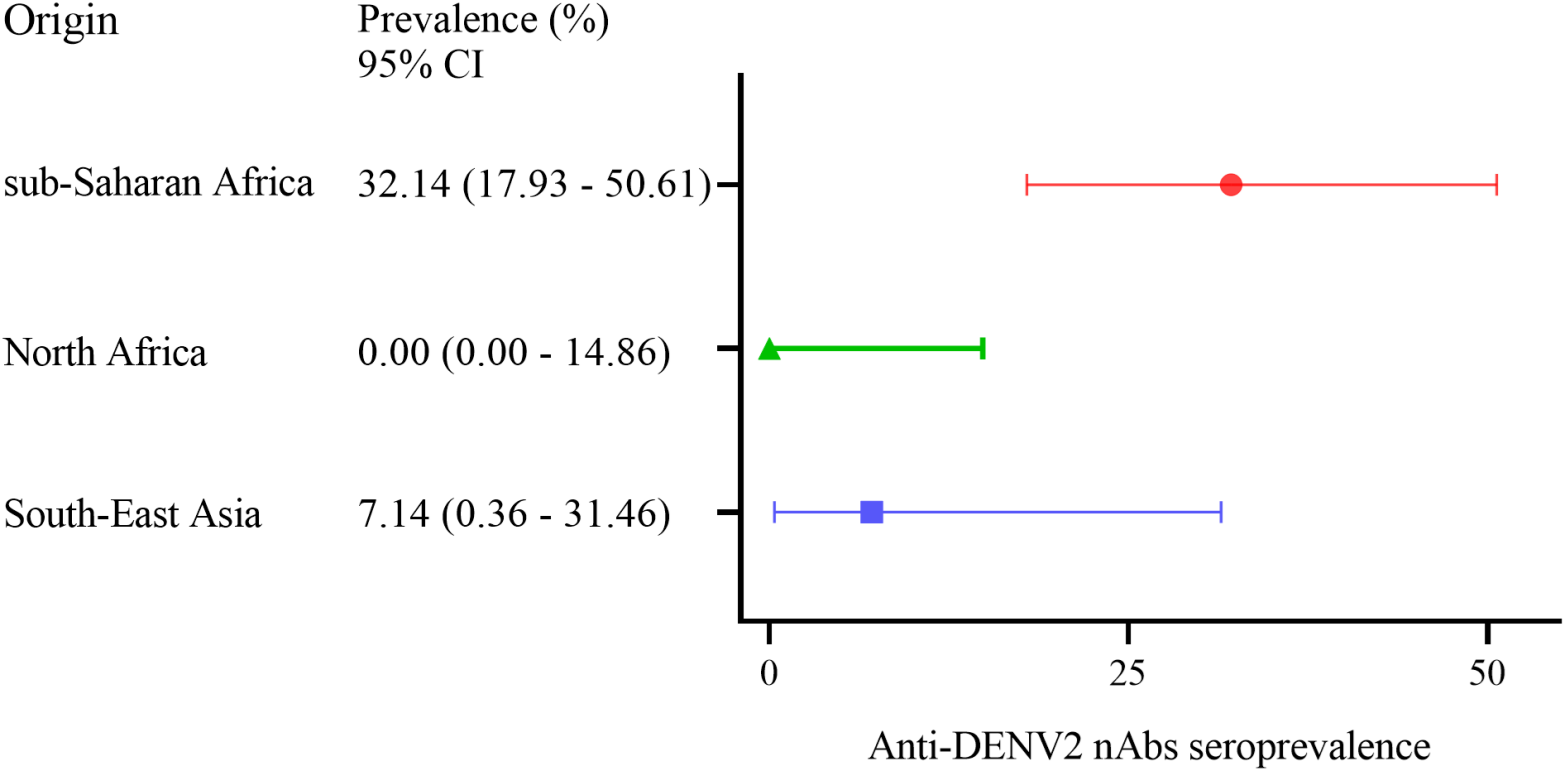
Forest plot for anti-DENV2 nAbs seroprevalence (%) measured by virus neutralization assay (VNT) among migrants arriving in Italy from sub-Saharan Africa (n=9/28), North Africa (n=0/22) and South-East Asia (n=1/14). Error bars indicate the 95% confidence interval (CI).

A total of 37 serum samples with IFA anti-DENV IgG titers and those with borderline anti-DENV IgM titers (n=2) were analyzed by virus neutralization assay (**Table 2**). We found the presence of anti-DENV2 nAbs [median dilution of anti-DENV2 nAbs: 1:40 (range: 1:20 - 1:160)] in 10 serum samples (n=10/37, 27%) that had previously shown anti-DENV IgG titer ≥1:40 tested by IFA (**Figure 3A**). In contrast, the two anti-DENV IgM positive sera, had anti-DENV2 nAbs titers <1:10. Univariable analysis showed that the frequency of anti-DENV2 nAbs varied by country of origin, being higher in sub-Saharan Africa (n=9/28, 32.14%) compared to South-East Asia (1/14, 7.14%) and North Africa (n=0/22, 0%) (p<0.01) (**Figure 4**). In contrast, age and gender were not associated with the development of anti-DENV2 nAbs in the study group. The multivariable analysis confirmed that origin from sub-Saharan Africa was the only characteristic independently associated with the development of anti-DENV2 nAbs (aOR 21.86; 95% CI 2.20-217.47).

### Anti-YFV, anti-WNV and anti-USUV neutralization test

Serum samples with anti-DENV2 nAbs titer <1:10 (n=27/37, 72.97%) were analyzed for the presence of anti-WNVL1, anti-YFV17D and anti-USUV nAbs. Almost all samples negative for anti-DENV2 nAbs but with detectable anti-DENV IgG titer ≥1:20 tested by IFA showed nAbs to another orthoflavivirus (n=25/27, 92.59%) (**Figure 3B**). In particular, 17 sera (n=17/25, 68%) had nAbs against WNV [median dilution of anti-WNV nAbs: 1:40 (1:20 - 1:320)], 7 serum samples (n=7/25, 28%) had nAbs against YFV17D [median dilution of anti-YFV17D nAbs: 1:20 (1:20 – 1:80)] and 1 serum sample (n=1/25, 4%) had nAbs against USUV (dilution of anti-USUV nAbs: 1:20). No anti-WNVL1, anti-YFV17D or anti-USUV nAbs were detected in the remaining 2 serum samples. Of the 2 serum samples with anti-DENV2 nAbs titer <1:10 but positive for anti-DENV IgM by IFA, one had anti-YFV17D nAbs titer at a serum dilution of 1:10 and the other was negative for anti-WNVL1, anti-YFV17D and anti-USUV nAbs.

To evaluate a possible cross-reactivity between nAbs against DENV2 and other orthoflaviviruses, anti-DENV2 nAbs positive sera (n=10) were further tested for the presence of anti-WNVL1, anti-YFV17D and anti-USUV nAbs. Two anti-DENV2 serum samples showed similar levels of anti-YF17D and anti-WNVL1 nAbs, respectively (**Figure 3A**).

## Discussion

The growing challenge posed by human pathogenic orthoflaviviruses in the 21st century is highlighted by the ongoing transmission of ZIKV across several countries (Rabe et al., 2025) and the epidemics of DENV around the world, including the recent outbreaks in several European countries (Frasca et al., 2024). In addition, connectivity between population centers is closely linked to the import/export of infectious diseases, both directly and through vector-borne diseases (Núñez-López et al., 2021; Wesolowski et al., 2017; Khan et al., 2014). Human migration for cultural or economic reasons plays an important role in the movement of susceptible and infectious individuals over long distances. This depends on the epidemiological profile of the countries of origin (Núñez-López et al., 2021). In this context, ZIKV and DENV are of particular concern as migration patterns may increase the risk of their spread (Wesolowski et al., 2017; Heesterbeek et al., 2015; Martinez-Vega et al., 2015), although data show a limited evidence on the risk of transmission of infectious diseases, such as tubercolosis, viral hepatitis, HIV, malaria, intestinal nematodes and arboviral infections, from migrants to host populations (Vignier and Bouchaud, 2018). In our study group, no viremic individuals were observed as indicated by the absence of ZIKV and DENV RNA detection in serum samples from migrants entering Italy from North and sub-Saharan Africa and South-East Asia, where these viruses are considered endemic (Asaga et al., 2023), suggesting no risk of virus transmission in the host country. However, as expected, our study confirms the high prevalence of DENV and other orthoflavivirus infections in migrants’ countries of origin. In general, the majority of migrants arriving in high-income countries originates from regions with a high prevalence of transmissible infectious diseases. Moreover, the risk and burden of infectious diseases are not evenly distributed among migrant groups and vary with the stage of migration. For example, the transmission dynamics of certain viral infections, such as DENV, are closely linked to human mobility and can be partially predicted using mobility models to generate dynamic risk maps (Núñez-López et al., 2021). It is therefore essential to implement early screening measures and to promote research aimed at assessing the real risk of importation of specific infectious diseases (Baker et al., 2024). According to our results, almost 30% (n=9/28) of sub-Saharan African migrants whose blood sample was taken on arrival in Italy in 2023, as indicated by their seropositivity for DENV-specific neutralizing antibodies, had been exposed to DENV at some point in their lives. In our study, we used DENV2 to determine anti-DENV nAb titers, due to its optimal growth characteristics in cell culture. This makes it a reliable and standardized tool for accurately assessing anti-DENV nAbs responses. Additionally, given the high degree of homology among the various DENV serotypes, any discrepancies in VNT outcomes are anticipated to be minimal. Several African countries have reported outbreaks caused by all four DENV serotypes (DENV1-4) (Eltom et al., 2021), with DENV2 responsible for most epidemics (Mwanyika et al., 2021). It is currently estimated that 16% of the annual global burden of DENV occurs in sub-Saharan Africa (Onoja et al., 2023). Nevertheless, the epidemiological situation in Africa is still uncertain and the reported incidence is low due to limited diagnostic testing, few hospitals and the misdiagnosis of other acute febrile illnesses such as malaria (Stoler et al., 2014). Some studies have also suggested that there may be some protection against severe dengue in people of African ancestry (de la C Sierra et al., 2007; Halstead et al., 2001; Oliveira et al., 2018; Boillant-Blanco et al., 2018), a condition that could eventually lead to an increased number of milder clinical presentations of dengue in Africa, often undiagnosed. In addition, the epidemiology of infections in Africa, including DENV, is highly complex and influenced by various factors such as the continent’s diverse environments, climatic zones and population densities (Nakase et al., 2024). As a result, data on DENV transmission in Africa often appear inconsistent and contradictory, reflecting the lack of reliable epidemiological information and the diversity of climates and environments. This confuses clinicians and hinders the development of effective interventions, perpetuating the cycle of inadequate control. Despite the limited sample size, our study has contributed to a better understanding of the epidemiology and seroprevalence of DENV in the sub-Saharan region. According to Italian vaccination guidelines, serological screening for DENV is not mandatory for this population. Similarly, most European countries, including Italy, do not have specific immunization programs for newly arrived migrants with a history of DENV infection, leaving migrants at risk of severe clinical manifestations of dengue in the case of a second infection. In Italy, all imported cases, including those involving travelers and newly arrived migrants, such as more than 400 imported DENV cases in 2024 (Del Manso et al., 2024), have a potential risk of secondary DENV infection due to the increase in autochthonous transmission outbreaks recorded in the country in recent years (Frasca et al., 2024; Del Manso et al., 2024). Indeed, while neutralizing antibodies produced during the first exposure confer lifetime protection against the same serotype, these antibodies cannot efficiently neutralize other DENV serotypes leading to the Antibody-Dependent Enhancement (ADE) phenomenon, that enhances viral internalization into cells and increases the risk of severe disease by 15 to 80 times (Guzman and Vazquez, 2010). Consequently, clinical history should be carefully considered prior to vaccine administration (da Silveira et al., 2019). Italian Medicines Agency (AIFA) has so far approved two DENV vaccines, Dengvaxia and Qdenga, with the latter being the only currently commercialized in Italy (Venturi et al., 2024). Qdenga (TAK-003) is a live attenuated tetravalent vaccine, based exclusively on DENV backbone and is able to confer protection against all DENV serotypes, therefore, is indicated to individuals from 4 years old even in the absence of a previous infection although is suggested only for people traveling to endemic areas or with dengue outbreaks (Venturi et al., 2024). Identifying target populations who could benefit from DENV vaccines should therefore be considered a key public health priority, particularly among the most vulnerable groups, such as newly arrived migrants, who often face significant barriers to accessing health services due to legal, economic and cultural challenges. Orthoflaviviruses are known to induce antibodies that may cross-react with closely related viruses, and this is further complicated by their co-circulation in endemic areas. This also applies to cross-reactive antibodies that may be induced by arbovirus vaccination, if available (Kasbergen et al., 2023). Due to the high antigenic similarity of some orthoflaviviruses, pre-existing antibodies may lead to false-positive results in serological tests, posing a challenge to diagnosis (Fischer et al., 2021). In our study, serum samples from migrants showed varying degrees of cross-reactivity between orthoflaviviruses. Approximately 60% of anti-DENV IgG positive sera, but negative for anti-DENV2 nAbs, showed anti-WNVL1 antibodies. In addition, almost 30% of anti-DENV IgG reactive samples had anti-YFV17D nAbs, suggesting previous exposure to YFV or YF vaccination rather than DENV, and only one sample was positive for anti-USUV nAbs. The extent of cross-reaction depends largely on the degree of antigenic similarity between orthoflaviviruses (Endale et al., 2021). The orthoflavivirus E protein, which plays an important role in virus receptor binding, host cell entry, and antigenicity (Li et al., 2016), contains three structurally distinct domains, DI, DII and DIII. The latter, which exclusively elicits potent virus-neutralizing antibodies, contains the B-cell epitope EXE/DPPFG, which is highly cross-reactive in most orthoflaviviruses. Multiple sequence alignment of the E protein among orthoflaviviruses revealed that this protein of DENV2 shares 54% amino acid homology with ZIKV (Endale et al., 2021). Similarly, the EXE/DPPFG epitope of the DIII domain is almost 85% conserved in DENV, ZIKV and WNV (Uhuami et al., 2024). To our knowledge, antibody cross-reactivity between DENV and USUV is less well characterized. We found a high correlation between the ELISA and IFA methods for detecting anti-DENV IgG (n=37/38, 97.36%) but a low level of concordance between the same serological assays for anti-ZIKV IgM (n=1/8, 12.50%) and anti-DENV IgM (n=2/10, 20%). These differences can be partly explained by the affinity maturation process of Ig antibodies. IgG are produced later in the course of an infection, through the somatic hypermutation and class-switch recombination of IgM-producing B cells (Law et al., 2019). IgG are monomeric and exhibit high affinity and specificity for their target antigens, enhancing the consistency of detection across different assays, including ELISA and IFA. In contrast, IgM are produced early in the immune response, exhibiting low-affinity antigen-binding sites, despite their pentameric structure conferring high avidity (Law et al., 2019). This lower intrinsic affinity can contribute to less consistent detection between serological assays. Moreover, the detection of IgM is complicated by substantial cross-reactivity among orthoflaviviruses (Griffin et al., 2019). A strength of our study is that it highlights the importance of combining two or more serological methods to improve the accuracy of orthoflavivirus diagnosis. Indeed, cross-reactive epitopes among orthoflaviviruses can lead to non-specific binding in serological assays, especially ELISA. This may account for false-positive results in regions where multiple orthoflaviviruses co-circulate or where vaccination against orthoflaviviruses (e.g., YFV) is common (Musso and Despres, 2020). Additionally, while IFA is highly specific, it is generally less sensitive than ELISA, particularly for low-titer antibodies (Khalifah et al., 2022; Copple et al., 2011). This reduced sensitivity may result in false negatives in cases where IgM levels are low but still detectable by ELISA. Therefore, weak IgM responses may be missed by IFA but detected by ELISA. Conversely, the broader reactivity of ELISA IgM detection may lead to false positives that are not confirmed by IFA. Nevertheless, for definitive diagnosis, serum samples reactive to IFA should be confirmed by the virus neutralization test, the “gold standard” confirmatory test for discriminating orthoflavivirus exposure. Indeed, the two serum samples that were confirmed by IFA to contain anti-ZIKV IgM and anti-DENV IgM showed neutralizing activity against WNVL1 and YF17D, respectively. This supports the hypothesis of cross-reactive or past heterologous orthoflavivirus exposure. Whether the presence of nAbs may interfere with the measurement of IgM in serological diagnosis has not been widely discussed. However, Houghton-Triviño et al., found anti-DENV IgM positive sera in individuals vaccinated against YFV, although none of them were positive prior to vaccination (Houghton-Triviño et al., 2008). The detection of anti-ZIKV IgM in a serum sample exhibiting anti-WNVL1 nAbs may be partly due to antigenic cross-reactivity between ZIKV and WNV. These viruses share approximately 57% amino acid sequence homology in their envelope (E) protein (Chan et al., 2022). The study had several limitations. First, the small number of cases included in the study limits the ability to provide a meaningful estimate of ZIKV and DENV prevalence in North Africa, sub-Saharan Africa and South-East Asia. In addition, the lack of virus neutralization testing against other orthoflaviviruses [i.e. Japanese encephalitis virus (JEV) and tick-borne encephalitis virus (TBEV)] or other arboviruses, such as those belonging to the *Togaviridae* family (e.g. Chikungunya virus), could limit the final interpretation of the results obtained from those serum samples that were positive for anti-DENV IgG or anti-DENV IgM but negative for nAbs to DENV2, WNVL1, YF17D and USUV. We also recognize that testing of paired serum samples is considered to be the most reliable sero-diagnostic method, with increasing titers being required for confirmation of diagnosis.

In conclusion, none of the migrant serum samples tested positive for the detection of ZIKV or DENV RNA. However, a significant number of the samples contained antibodies to DENV and other emerging orthoflaviviruses (50% in total), which are associated with previous infections. This confirms the circulation of these viruses in the migrants’ countries of origin, particularly in sub-Saharan Africa. This underlines the importance of continued public health surveillance, as well as the need to implement risk communication measures and additional screening programmes tailored to emerging orthoflaviviruses. From a diagnostic perspective, the global spread of ZIKV, DENV and other orthoflaviviruses emphasizes the importance of considering how cross-reactive immunity can affect the diagnosis of these emerging mosquitoes borne viral infections. A key implication of our findings is that, in some cases where antibodies are present, infections may be misclassified as confirmed orthoflavivirus positivity if testing for other known circulating arboviruses or available vaccines is not conducted in parallel. Our study demonstrates how the serological diagnosis of orthoflaviviruses is complicated by an individual’s history of cross-reactive antibody production in the context of the global co-circulation of antigenically related arboviruses.

## Data Availability

The raw data supporting the conclusions of this article will be made available by the authors, without undue reservation.
